# Effect of pH and Heat Treatment on the Antioxidant Activity of Egg White Protein-Derived Peptides after Simulated In-Vitro Gastrointestinal Digestion

**DOI:** 10.3390/antiox9111114

**Published:** 2020-11-11

**Authors:** Priyanka Singh Rao, Emerson Nolasco, Akihiro Handa, Michael J. Naldrett, Sophie Alvarez, Kaustav Majumder

**Affiliations:** 1 Dairy Chemistry Division, National Dairy Research Institute (ICAR-NDRI), Karnal, Haryana 132001, India; priyankasinghrao@gmail.com; 2 Department of Food Science and Technology, University of Nebraska-Lincoln, Lincoln, NE 68588-6205, USA; enolasco2@huskers.unl.edu; 3 Institute of Technology Solution, R&D Division, Kewpie Corporation, 2-5-7 Sengawa, Tokyo 1820002, Japan; akihiro_handa@kewpie.co.jp; 4 Proteomics and Metabolomics Facility, Nebraska Center for Biotechnology, University of Nebraska-Lincoln, Lincoln, NE 68588-6205, USA; mnaldrett@unl.edu (M.J.N.); salvarez@unl.edu (S.A.)

**Keywords:** egg white powder, heat treatment, protein aggregation, simulated digestion, antioxidant activity, peptide profile

## Abstract

The study aimed to analyze pH and heat treatment’s effect in modulating the release of peptides with antioxidant activity after simulated gastrointestinal (GI) digestion of Egg white powder (EWP). EWP samples with neutral (EWPN) and alkaline (EWPA) pH were heat-treated at 20, 60, and 90 °C and analyzed for protein aggregation, solubility, and GI digestibility. Heat treatment decreased solubility and induced protein aggregation, which was higher for EWPN as compared to EWPA. The unfolding of EWPA proteins at 60 °C exhibited a higher GI digestibility and antioxidant activity via Oxygen Radical Absorbance Capacity (ORAC) assay as compared to EWPN. Interestingly, a reverse trend was observed in the cellular antioxidant assay, and the GI-digest of EWPN exhibited a higher antioxidant activity. The LC-MS/MS analysis are in concordance with cellular antioxidant activity assay and showed a higher intensity for peptides with potential antioxidant activity in the GI-digest of EWPN. The results indicate that heat treatment but not the pH is a critical factor in improving the protein digestibility and releasing peptides with antioxidant activity after GI digestion.

## 1. Introduction

Chicken eggs are an essential part of the human diet. Eggs are nutritious, economical, accessible, and utilized in a wide range of food preparations globally. A 16 percent increase in per capita egg consumption has been observed in the United States over the past 20 years. Moreover, the use of egg products, especially the dried egg white powder, has increased substantially [1]. The whole egg is composed mainly of egg white (63%) and egg yolk (27.5%) [2]. In a whole egg, egg white proteins have shown high gastrointestinal (GI) digestibility, thus recognized as a critical dietary source of essential amino acids vital for human growth and development [3]. 

De-sugared and dried egg white powder are primarily used in the food industries due to the convenience of storage and transport [4]. Egg white’s excellent functional properties such as emulsification, gelation, and foaming properties make the egg white powder a valuable ingredient for different food preparations [5]. Nonetheless, egg white proteins’ functionalities are substantially affected by temperature and pH, either individually or in combination [6,7]. 

Heat treatment denatures egg white proteins [8,9]. The denaturation process of egg white proteins also depends on various other factors, such as pH [10,11]. The pH and temperature-dependent protein unfolding forms the intermolecular linkage and causes aggregation [11,12]. Heat or pH-induced changes in the protein conformation influence the protein aggregation and could modulate the enzymes’ accessibility and the digestibility of the egg white proteins [9,13,14,15].

Previous studies have shown that ingested food proteins could release bioactive peptides after GI digestion and exhibit different health-beneficial physiological functions after absorption [13,16,17,18]. Studies have also reported that spray-dried egg white possesses antioxidant properties after being subjected to pepsin digestion [19]. However, the effect of heat and pH during egg white processing and its impact on modulating the GI digestibility and production of bioactive peptides has remained unexplored. Such information will facilitate the use of egg white powder in several products in the food industry [4]. Overall, minimal information is available about the effect of pH and heat treatment and their subsequent effect in producing bioactive peptides from egg white protein. Thus, it is crucial to determine how factors such as pH and heat treatment influence the release of bioactive peptides from egg white powder after GI digestion to facilitate the use of egg white as a functional food ingredient. 

Thus, the current work aims to analyze the effect of pH and temperature in modulating the release of peptides with antioxidant activity upon GI digestion of egg white powder.

## 2. Materials and Methods

### 2.1. Materials

Two different spray-dried egg white powders (EWP) with neutral pH (6.8) and alkaline pH (9.1) were obtained from Henningsen Foods, Inc. (Omaha, NE, USA). The porcine bile extracts (B8631), pepsin from porcine gastric mucosa (P7012), and pancreatin from porcine pancreas (P1750) were purchased from Sigma Aldrich (St. Louis, MO, USA). α-amylase from *Bacillus subtilis* (0210044725) was purchased from MP Biomedical (Irvine, CA, USA). If not stated, all other chemicals were obtained from Millipore Sigma (St. Louis, MO, USA). 

### 2.2. Temperature Treatment of Egg White Powder (EWP) 

The neutral pH EWP (EWPN) and alkaline pH EWP (EWPA) samples were dissolved (5% w/v) in distilled water. Solutions were heat-treated at 60 and 90 °C in a shaking water bath (Thermo Fisher, Waltham, MA, USA) for 10 min. Heat-treated samples, along with a control sample (without heat treatment, i.e., room temperature ~20 °C), were then centrifuged at 4600× *g* for 20 min at 20 °C (Thermo Fisher Sorvall Legend X1R, Waltham, MA, USA). The obtained supernatant was freeze-dried (LabconcoFreeZone 6L, Kansas City, MO, USA) and stored at −80 °C until further analysis.

### 2.3. Protein Content and Solubility 

Lowry’s method was used to measure the freeze-dried EWP samples’ protein content from three different heat treatment groups [20]. The complex-forming reagent (2% Sodium carbonate: 1% CuSO_4_: 2% Sodium potassium tartrate; 100:0.5:0.5) and clear sample (protein concentration ≤ 1000 μg/mL) were mixed thoroughly in 1:10 ratio. After 10 min of incubation at 37 °C, the sample volume equivalent Folin’s reagent (0.1 N) was added, mixed properly, and incubated for 30 min. The developed color was measured using an 800 TS microplate reader (Biotek, Winooski, VA, USA) at 750nm wavelength. The solubility was calculated as the percentage of the protein content in the treated sample to that of the control sample’s corresponding protein content (20 °C). For clarity purposes, the control group (the room temperature treated group) is classified as “20 °C” treatment.

### 2.4. Sodium Dodecyl Sulfate-Polyacrylamide Gel Electrophoresis (SDS-PAGE)

The effect of heat treatment on the egg white protein sample was assessed by SDS-PAGE analysis. Freeze-dried EWP samples were mixed in a 1:1 ratio with Laemmli sample buffer to obtain a 1 mg/mL protein solution (Bio-Rad, Hercules, CA, USA.) heated at 100 °C for 5 min. Subsequently, 20 μL from each sample was loaded to a polyacrylamide gel’s wells to obtain a 20 μg protein concentration per well (4–20% Tris-Glycine precast polyacrylamide gel, 10 well, 50 µL, Bio-Rad, Hercules, CA, USA). Precision plus protein dual-color standards (Bio-Rad, product No. 161-0374) were used as molecular weight markers. The voltage was set at 100 V for 1.5 h. The gels were dyed using Coomassie brilliant blue R-250 for 10 min followed by de-staining (Methanol: glacial acetic acid: distilled water (20: 10: 70, (v/v/v))). Gel images were captured by Odyssey CLx imaging system (LI-COR, Lincoln, NE, USA).

### 2.5. Simulated In-Vitro Digestion of Egg White Powder

In vitro GI digestion of egg white powder sample was performed by following the INFOGEST protocol with minor modifications [21]. Freeze-dried EWP samples (5% w/v in water, dry weight) were digested for three major phases: oral, gastric, and intestinal. The simulated salivary fluid (SSF), simulated gastric fluid (SGF), and simulated intestinal fluid (SIF) were prepared as detailed in Minekus et al. 2014 [21]. Details of SSF, SGF, and SIF composition is given in Table S1. First, oral digestion was performed by mixing the sample volume in a 1:1 ratio with SSF, α-amylase enzyme (75 U/mL in the final oral bolus volume), 0.3 M CaCl_2_ (50 µL), and water (1.95 mL) in a stomacher bag (Seward; BA6040/CLR) and stomached for 2 min in a Seward Stomacher^®^ 80 (West Sussex, United Kingdom). For the gastric phase, the 1:1 ratio was maintained by adding SGF, 0.3 M CaCl_2_ (10 µL), and water (1.39 mL) to the oral bolus in a 150 mL jacketed beaker, which was maintained at 37 °C through constant water circulation. The pH was adjusted to 3.0 using 6 M HCl, and the sample was digested by pepsin (2000 U/mL in the final gastric chyme volume) for 2 h. Afterward, the pH was increased to 7.0 to inactivate the enzyme by adding 6 M NaOH solution. Finally, gastric chyme was mixed with SIF, (0.3M) CaCl_2_, water, bile salt solution (10 mM concentration in final intestinal volume) in a 1:1 ratio and subjected to further intestinal digestion by pancreatin (100 U/mL in final intestinal volume) for another 2 h. The pH during gastric and intestinal hydrolysis was maintained using a Titrando 902 with constant stirring by Stirrer 801 (Metrohm AG, Herisau, Switzerland). The hydrolysis was terminated by lowering the pH to 6.0 by adding 6 M HCl; the digested samples were frozen and freeze-dried for further analysis. Each sample was prepared individually, and the digestion was performed in triplicate. The composition of the buffers used in the gastrointestinal digestion was illustrated in the Table S1.

The degree of hydrolysis was analyzed for each digested sample by the pH-stat method using the following formula:

Equation (1). Degree of hydrolysis of gastric digestion.

(1) $$DH_{protein}=100\times \frac{V\left(HCl\right)\times N\left(HCl\right)}{\left(1-\alpha \left(COOH\right)\right)\times m\left(protein\right)\times h_{tot}}$$

Equation (2). Degree of hydrolysis of intestinal digestion.

(2) $$DH_{protein}=100\times \frac{V\left(NaOH\right)\times N\left(NaOH\right)}{\alpha \left(NH_{2}\right)\times m\left(protein\right)\times h_{tot}}$$

where *V* is the acid or alkali consumption in mL, *N* is the normality of acid or alkali, *α* the average degree of dissociation of the α-NH_2_ groups, m is the mass of protein being hydrolyzed (g), and *h_tot_* is the total number of peptide bonds in the protein substrate (meqv/g protein). The *h_tot_* for egg white hydrolysates is 7.67 meqv per g protein [22].

### 2.6. Oxygen Radical Absorbance Capacity (ORAC) Assay

The ORAC assay was performed as described by Huang et al., 2010, with modifications [23]. Using a 96-well plate, 100 µL of Trolox standards (0.01–0.03 mM), EWP hydrolysate samples, or phosphate buffer (pH 7.4, as blank) were added in different wells. Next, 50 µL of fluorescein (200 nM) was added to each well and incubated within the equipment at 37 °C for 15 min, followed by the addition of 50 µL of 2,2-Azobis(2-methylpropionamidine) dihydrochloride (AAPH) (80 mM) to each well using Synergy H1 automatic dispenser for a total volume of 200 µL. In two separate wells, 100 µL of AAPH at 80 mM and 50 µL of 200 nM fluorescein were separately added to confirm the absence of fluorescence from AAPH and stability of fluorescein throughout the process, respectively (data not shown). The fluorescence reading was recorded up to 70 min at one-minute intervals for excitation and emission wavelengths of 485 and 538 nm, respectively, using the fluorescent monochromators of the Synergy H4 Hybrid (Biotek, Winooski, VT, USA) Multi-Mode Microplate Reader.

### 2.7. Cell Culture

Gastrointestinal epithelial cells (Caco-2: ATCC^®^ HTB-37™, Manassas, VA, USA) were grown in Eagle’s Minimum Essential Medium (EMEM) (ATCC^®^ 30-2003™, Manassas, VA, USA) supplemented with 1% Penicillin-Streptomycin (Gibco, 15140122, Waltham, MA, USA) cocktail (v/v) and 20% fetal bovine serum (v/v) (FBS: Gibco, 10437028, Waltham, MA, USA) at 37 °C and 5% CO_2_ in a humidified condition. Cell media was replaced every 2 days. The cells were grown for 6–7 days to reach 70–80% confluency and then used for experiments. Cells between 22–25 passages were used in this study.

### 2.8. Reactive Oxygen Species (ROS) Measurement in Gastrointestinal Epithelial Cells

Caco-2 cells, seeded at a density of 25,000 cells/well in a 48 well TPP tissue culture plates (MidSci, MO, USA), were grown in EMEM media supplemented with 20% FBS (v/v) and 1% Penicillin-Streptomycin cocktail (v/v) at 37 °C containing 5% CO_2_ under humidified conditions. The cells were grown to near 90–95% confluency (for 5–6 days). After reaching confluency, the cells were washed with Dulbecco’s phosphate-buffered saline (DPBS) and kept for synchronization for 16–18 h in the basal medium of EMEM supplemented with 1% FBS (v/v) and 1% Penicillin-Streptomycin cocktail (v/v). After synchronization, the cells were treated and incubated with the EWP hydrolysates dissolved in basal media at 5, 10, and 50 μg/mL, based on the well volume, for 4 h. After the hydrolysate pretreatment, oxidative stress was induced in the cells by adding 10 ng/mL of tumor necrosis factor-alpha (TNF-α) for 30 min at 37 °C. After 30 min of incubation with TNF-α, the cells were then exposed to 10 μM of the oxidant-sensitive fluorescent probe dihydroethidium (DHE) for 20 min at 37 °C and immediately observed using EVOS^®^ FL Auto Cell Imaging System with a 10× magnification. Images were taken for each well and used to calculate the mean fluorescent intensity.

The images were then processed by ImageJ (NIH, Sacramento, CA, USA). Multiple sections were created for each image by keeping the height and width constant. Only the X and Y coordinates were changed to obtain multiple sections of the image. At least five coordinates were selected for each image. Once a section of the image was selected, the fluorescent intensity was calculated, and the obtained values were imported. The same section was used to count the total cell number. The image was inverted and transformed into an 8-bit image; the threshold was adjusted so that the maximum number of cells was visible. The outline of the particles was analyzed, and the obtained values were imported for further analysis. The mean fluorescent intensity (MFI) was calculated by dividing the obtained fluorescent intensity by the total number of cells for each section. The average MFI for all the sections was taken and analyzed using Graphpad Prism software (San Diego, CA, USA).

### 2.9. Peptide Profile of In Vitro Digested EWP through LC-MS/MS

An aliquot of the dried EWP hydrolysate (3 kDa permeate at 60 °C) was suspended (20 µg/µL) in distilled water. Hydrophilic Interaction Liquid Chromatography (HILIC) was performed to separate smaller peptides (2–3 amino acids) using a BEH-Amide 1.7 µm (2.1 × 100 mm, Waters) column and a Vanquish High-Performance Liquid Chromatograph (HPLC) (Thermo Fisher, Waltham, MA, USA) at 40 °C, the samples were further diluted ten times before injection. A flow rate of 300 µL/min was used with a gradient of A (0.1% formic acid in 100% LC-MS grade water) and B (0.1% formic acid in 100% acetonitrile) as follow: 90% B for 2 min, 90% to 40% B in 12 min, back to 90% B in 1 min. The data was acquired on a Q Exactive™ HF mass spectrometer (MS) (Thermo Fisher, Waltham, MA, USA) using a mass range of 60 to 750 *m/z* on single charged ions. The isolated ions were further fragmented using an isolation window of 2 *m/z.* The acquired data were analyzed using PEAKS studio (Bioinformatics Solutions Inc., Waterloo, Canada) to perform de novo sequencing of the chromatograms and integrate the peaks for quantification.

For the separation of the larger peptides (>4 amino acids) through Reverse Phase Chromatography (RPC), samples were diluted 200 times and run using an online peptide separation by first desalting peptides on a trapping column (C18 Pepmap100 0.3 × 5 mm, 5 µm, 100 Å) at 5 µL/min in 1% acetonitrile and 0.1% formic acid. Next, separation into the mass spectrometer was done using a 75 µm × 25 cm peptide CSH C18 130 Å, 1.7 µm nano-column (Waters), and running a linear gradient run at 260 nL/min from 5% B to 32% B over 36 min. Solvents: A is 0.1% formic acid in LC-MS grade water, and B is 0.1% formic acid in 80% acetonitrile. The Q Exactive™ HF MS was run in a data-dependent acquisition mode triggering on peptides with charge states 1 to 4 over the mass range of 375–1500 *m/z*. All MS/MS samples were analyzed using PEAKS studio and set up to search the UniProt-ref_prot_Gallus_gallus_UP000000539 database (20190617, 27,804 entries). Peptides were searched with a fragment ion mass tolerance of 0.020 Da and a parent ion error tolerance of 5.0 ppm.

Further analysis of the data obtained from the LC-MS/MS was performed to obtain a Venn diagram concerning unique and common peptides using Genevenn software (http://genevenn.sourceforge.net/). Progenesis QI was used for data standardization to perform a principal component analysis (PCA) based on the variables of the peptide’s intensity between the samples for the peptides identified under the HILIC only. As the analysis had a descriptive purpose, as no replicates were performed.

### 2.10. Statistical Analysis

Samples were analyzed in triplicate for all the parameters unless specified otherwise. Each data set was first checked for normal distribution by the Shapiro-Wilk test. The normally distributed data were analyzed by ANOVA using GraphPad Prism (version 8.0.1; San Diego, CA, USA). Means were represented as the mean ± standard error of the mean (SEM). One-way or two-way ANOVA determined the statistical significance of the data, followed by Tukey’s or Bonferroni’s multiple comparisons tests, wherever applicable, with a *p*  <  0.05 taken as the value of significance.

## 3. Result and Discussion

### 3.1. Effect of Heat Treatment on Protein Content and Solubility of Egg White Protein(EWP)

**Figure 1:**
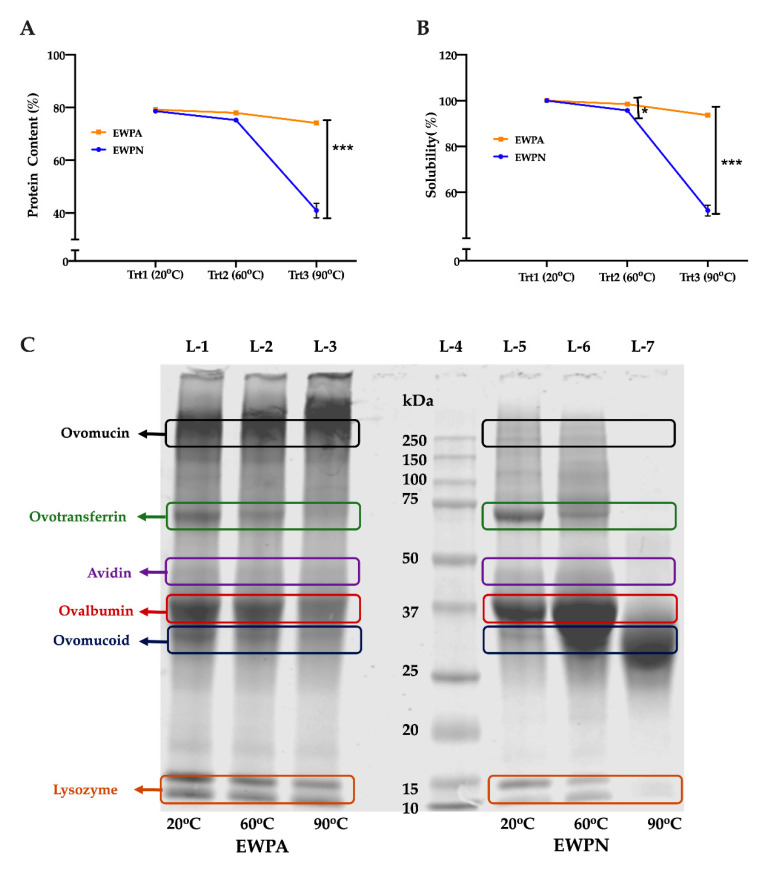
Effect of heat treatment on protein content and solubility of egg white protein samples. (**A**) Protein content and (**B**) Solubility of the Egg White Powder Neutral (EWPN) samples significantly decrease after heat treatment compared to Egg White Powder Alkaline (EWPA). The treatment groups are, treatment1 (Trt1) 20 °C, treatment2 (Trt2) 60 °C, treatment3 (Trt3) 90 °C. Two-way ANOVA followed by Bonferroni’s multiple comparison post hoc test was used to determine the significant difference between different samples, * and *** indicate *p* < 0.05 and *p* < 0.001, respectively. (**C**) Sodium Dodecyl Sulfate-Polyacrylamide Gel Electrophoresis (SDS-PAGE) analysis of egg white powder (EWP) samples indicates the denaturation of egg white protein Ovotransferrin and Ovalbumin; the denaturation is more prominent in the EWPN sample after heat treatment at 90 °C.

To assess the effect of pH and heat treatment, a 5% solution of the EWPN and EWPA samples were heat-treated (20, 60, and 90 °C) and analyzed for protein content and solubility. EWPN showed a significant decrease (*p* < 0.001) in the protein content and solubility after heat treatment at 90 °C (Figure 1A,B). Furthermore, the protein solubility, but not the protein content for the EWPN samples was significantly decreased after heat treatment at 60 °C. The observed phenomena indicate that the egg proteins remain relatively more stable at alkaline conditions.

SDS-PAGE was performed to evaluate the effect of heating on egg white protein aggregation, and the electropherogram is presented in Figure 1C. Visible changes were observed in the egg white protein fractions bands as the heating temperature increased from 20 to 90 °C, indicating the denaturation and aggregation of egg white proteins ovalbumin, ovotransferin, and lysozyme fractions.

Heating of egg white protein resulted in the formation of soluble and insoluble aggregates. Insoluble aggregates of ovotransferrin (~75 kDa), ovalbumin (~37 kDa), and lysozyme (~14 kDa) were formed, and the band intensity faded at 90 °C. A fade band around 50kDa was observed, which might be Avidin [24]; however, further study needs to be performed for confirmation.

The stability of these proteins depends on disulfide bonds [25]. The observation further indicates that the alkaline pH of the EWPA sample induced less aggregation of egg proteins than EWPN. However, the ovotransferrin band (~75kDa) faded significantly in EWPN and EWPA samples, especially after the heat treatment at 90 °C. Meanwhile, the intensity of the ovomucoid protein bands (aggregation) increased in sample EWPN at 90 °C. Previous findings support the results of this study. Ovomucoid, the most heat resistant protein in egg white, formed soluble aggregates and could sustain 100 °C heat treatment for at least 30 min [26,27].

The effect of heating on the proteins’ aggregation was more significant for EWPN than in the EWPA group, which agrees with the protein content and solubility (Figure 1A,B). Our results agree with the observation of [28] and [29], suggesting less aggregation upon heat treatment of egg white at pH 9–9.5 due to increased intermolecular repulsion of egg white proteins alkaline pH. The heterogeneous protein mixture in egg white gets gradually denatured upon heating, and the stability of these proteins is dependent on pH [7,10]. Thus, pH and heat treatment can modulate the protein structure and subsequently affect the enzymes’ accessibility, protein digestibility, and the release of peptides with potential biological activities.

### 3.2. Degree of Hydrolysis (DH) of Simulated Digested EWP

**Figure 2:**
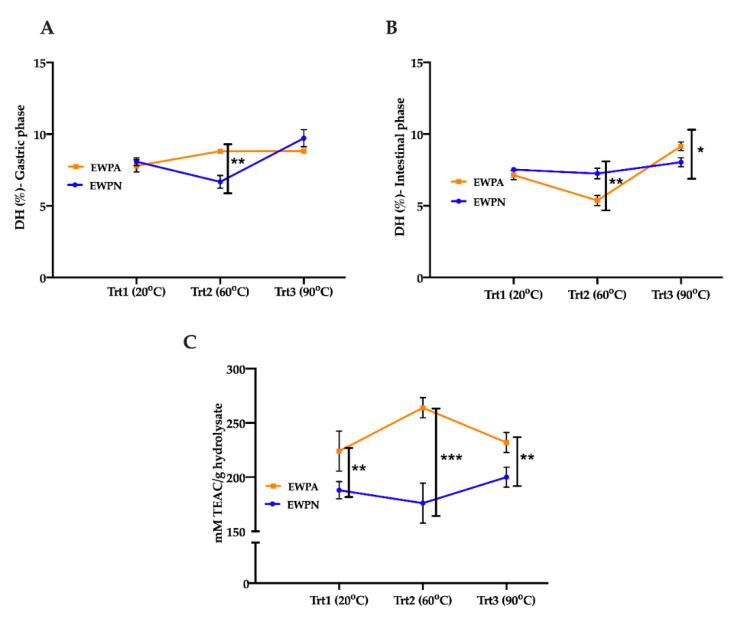
Degree of hydrolysis and in vitro antioxidant activity of egg white powder hydrolysate at different temperatures; (**A**) Gastric phase digestion, (**B**) Intestinal phase digestion, and (**C**) Antioxidant activity of egg white powder hydrolysate at different temperatures. Error bars represent the standard deviation (mean ± SD). Two-way ANOVA followed by Bonferroni’s multiple comparison post hoc test was used to determine the significant difference between different samples, *, **, and *** indicate *p* < 0.05, *p* < 0.01, and *p* < 0.001, respectively. EWPN: Egg White Powder Neutral, EWPA: Egg White Powder Alkaline.

The DH of EWP samples was determined after simulated GI digestion by the pH-stat method. The gastric and intestinal DH of EWP samples obtained via in vitro simulated digestion is shown in Figure 2. Under gastric conditions (Figure 2A), EWPA heated at 60 °C had a higher digestibility than EWPN, which might be due to the partial denaturation of egg white proteins in EWPA. The results correspond with the study of [6] and [7] in which denaturation of egg white protein with increased surface hydrophobicity was observed at alkaline pH at 65 °C. EWPN aggregates were formed at 60 °C (Figure 1C), which may hinder the gastric enzyme’s accessibility, pepsin, to its respective cleavage sites resulting in a lower DH. 

The DH results obtained after the intestinal phase digestion of EWPA and EWPN are presented in Figure 2B. The DH values obtained for the intestinal phase at 60 °C have a reverse trend compared to the gastric phase, which validates that EWPA, with partial denaturation at 60 °C, digested better at a lower pH during the gastric phase. Thus, a low availability of protein or peptides with accessible active sites for proteolysis during the intestinal phase, resulting in a lower DH value for EWPA.

Similarly, EWPN has an opposite trend due to the aggregation of proteins at 60 °C. A higher intestinal DH was observed at 90 °C for both samples, and the DH value of EWPA was significantly higher. Higher protein denaturation and unfolding at 90 °C could increase the intestinal enzyme’s accessibility to its cleavage sites, leading to a DH increase [28].

### 3.3. Antioxidant Properties of In Vitro Digested EWp by ORAC Assay

The variation in the antioxidant activity in the heat-treated and digested EWPN and EWPA samples was determined through the ORAC assay and reported in Figure 2C. Both egg white powders showed oxygen radical scavenging activity after digestion without the heat treatment (corresponding to the temperature 20 °C), but with some differences. However, EWPA had a significantly higher radical scavenging activity than EWPN (*p* < 0.01) irrespective of the heat treatment temperature. EWPA showed the highest radical scavenging activity after heat treatment at 60 °C.

Based on results related to solubility, DH, and antioxidant activity by the ORAC assay, the hydrolysates of egg white powder with heat treatment at 60 °C for 10 min was selected for further characterization. The results also indicate that 60 °C heat treatment of EWPA can result in peptides with high antioxidant activity; thus, the following study aims to further evaluate the antioxidant activity through an in vitro cell model and identify the differences in peptide composition through LC-MS/MS analysis.

### 3.4. Effect of EWP Hydrolysates on TNF Alpha-Induced Superoxide Generation

**Figure 3:**
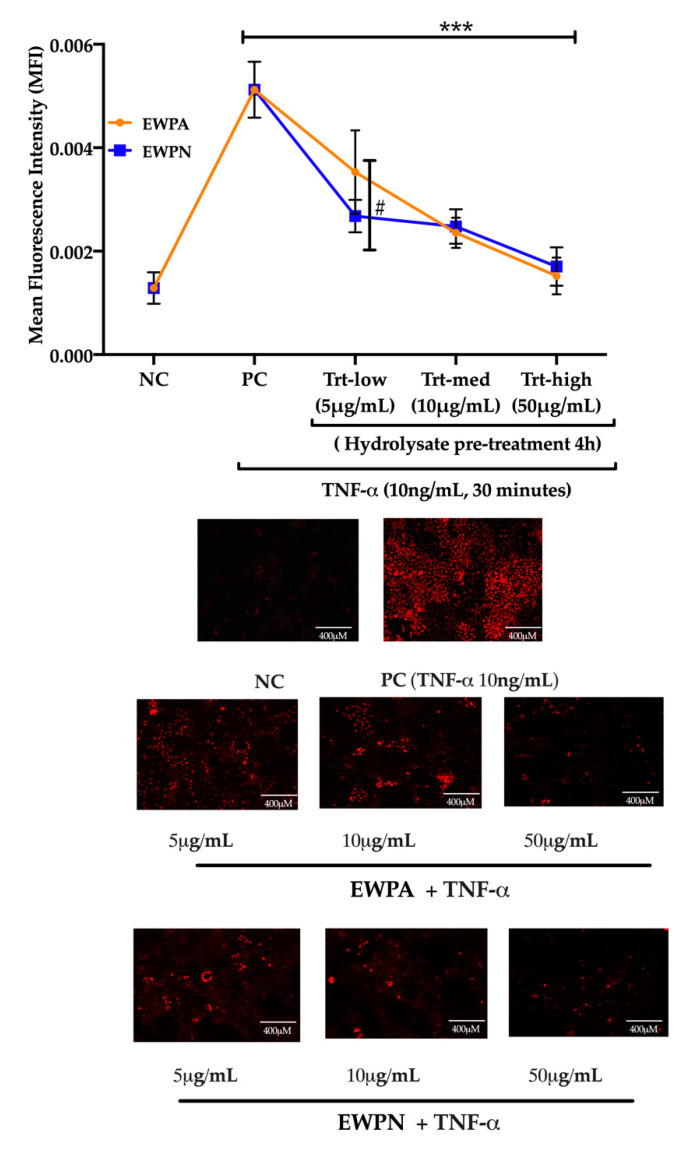
Effect of EWP hydrolysates on tumor necrosis factor-alpha (TNF-α) induced cellular reactive oxygen species (ROS). Both Egg White Powder Neutral (EWPN) and Egg White Powder Alkaline (EWPA) hydrolysate reduced TNF-αinduced ROS in gastrointestinal Caco2 cells. Error bars represent the standard deviation (mean ± SD, *n* = 5), two-way ANOVA followed by Bonferroni’s multiple comparison post hoc test was used to determine the significant difference between different samples, *** indicates *p* < 0.001. TNF-α induction (Positive control: PC) significantly increases ROS production compare to Negative control (NC). Three different treatment dosages, Treatment-low (Trt-low: 5 μg/mL), Treatment-medium (Trt-med: 10 μg/mL), Treatment-high (Trt-high: 50 μg/mL) were used, and all dosages for both samples (EWPA and EWPN) significantly reduced the production of ROS when compared to the positive control. Comparison between EWPA and EWPN at different dosages indicates that only at 5 μg/mL dosage EWPN exhibit significantly (# represents *p* < 0.05) stronger ROS inhibition compared to EWPA. Data were calculated as mean fluorescent intensity (MFI)/cell number. Scale bar: 400 μM (10× magnification).

The EWP GI hydrolysate’s effectiveness in reducing the generation of ROS was evaluated in intestinal epithelial cells. Three different concentrations of the hydrolysate were used in the study: treatment-low (Trt-low: 5 μg/mL), treatment-medium (Trt-med: 10 μg/mL), and treatment-high (Trt-high: 50 μg/mL). The method accuracy in determining the ROS inhibition using the DHE probe has been previously addressed in endothelial and vascular smooth muscle cells using ovotransferrin derived peptides and cooked eggs hydrolysates, respectively [29,30]. Our results indicate that the addition of egg white hydrolysate could significantly (*p* < 0.0001) reduce the ROS generation at all concentrations when compared to the positive control (PC) (Figure 3).

Peptides from EWP at a concentration of 50 μg/mL reduced the ROS production in Caco-2 cells. There was no significant difference between 10 and 50 μg/mL groups for EWPA and EWPN. However, EWPN hydrolysate exhibited a significantly stronger ROS inhibitory effect than EWPA hydrolysate in 5 μg/mL. Future studies should consider the effect of the EWP hydrolysate concentration on the Caco-2 cells proliferation by evaluating mitochondrial respiration through an MTT assay. This information would provide valuable insight into the long-term exposure of endothelial cells to the EWP hydrolysate.

The present study demonstrated the peptides’ antioxidant effect derived from the GI hydrolysate of heat-treated (60 °C) EWPA and EWPN samples, with minimum differences between these two samples. Interestingly, these results are very much different from the in vitro ORAC results. There could be several possible reasons for these differences. ORAC primarily measures the oxygen radical scavenging capacity [31], whereas TNF-α signaling enables the production of superoxide (O^−^) through the activation of the membrane-bound nicotinamide adenine dinucleotide phosphate (NADPH Nox2/gp91) oxidase [32]. The bioactive peptides derived from egg proteins had shown various biological activities like antioxidant, anti-inflammatory, angiotensin-I-converting enzyme (ACE) inhibitory (antihypertensive), antimicrobial, antidiabetic, and iron-/calcium-binding activities [33,34]. Numerous studies have shown that the peptides derived from egg white proteins possess an excellent antioxidant capacity. Egg protein ovotransferrin-derived peptides (IRW and IQW) has been shown to reduce oxidative stress in endothelial cells [35].

Furthermore, two peptides, AEERYP and DEDTQAMP (Ala-Glu-Glu-Arg-Tyr-Pro and Asp-Glu-Asp-Thr-Gln-Ala-Met-Pro), derived from egg white protein have also been reported to show excellent antioxidant properties [36]. Previous results also showed the differences between ORAC value and the egg white protein-derived peptides’ cellular antioxidant activity. Jahandideh et al., 2016 showed that antioxidant peptides earlier identified from egg white protein ovotransferrin through ORAC assay failed to exhibit similar activity in vascular endothelial cells [30]. In the present study, one of the reasons EWPA had higher antioxidant activity in ORAC could be related to the higher percentage of peptides with high molecular weight complying with the antioxidant constraints, containing one or more amino acids with radical quenching capacity. These results suggest that although the heat treatment changes soluble protein content and GI digestibility, it may not affect the release of bioactive peptides with antioxidant activity. Therefore, it is essential to identify and characterize the peptides presents in both EWPA and EWPA hydrolysates to delineate the reasons behind the observed biological activities.

### 3.5. Identification and Characterization of Peptides in the EWP Hydrolysates

The next step was to identify and characterize the peptides present in the GI hydrolysate of heat-treated (60 °C) EWPA and EWPN samples to comprehend the antioxidant activity. The peptides’ sequences were subjected to structural requirement constraints of antioxidant peptides based on previous literature. The constraints were in the presence of Tyr/Trp or Leu/Phe/Ile in the amino-terminal with the presence of Lys, Leu, His, Trp, Met, Tyr, and Glu in any position at the carboxylic end [37,38,39,40]. Both conditions need to be met to be counted as a potential peptide with antioxidant activity.

**Figure 4:**
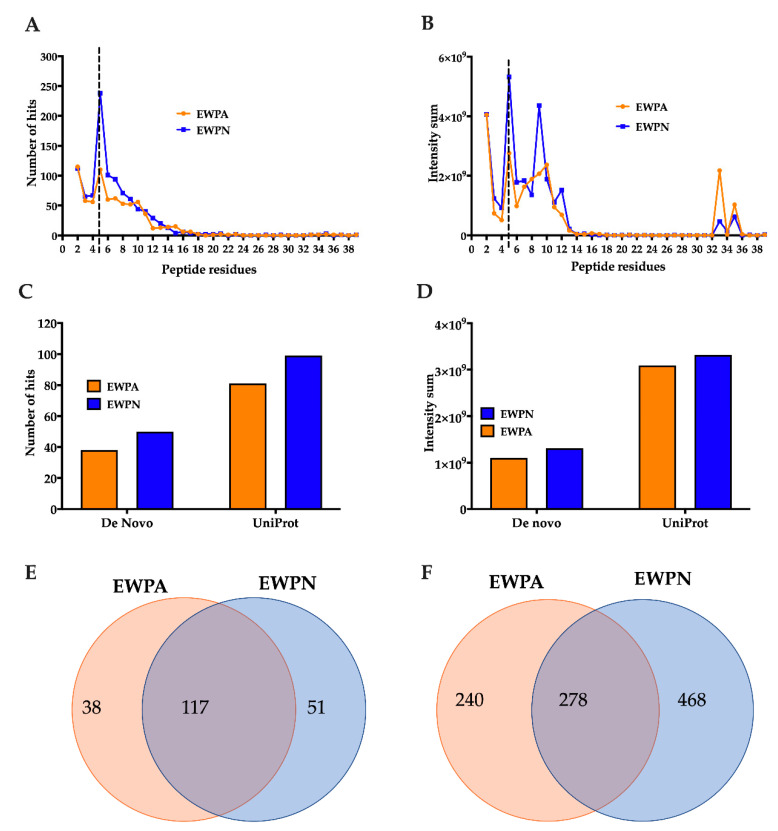
Peptide profile of EWPN and EWPA hydrolysate after LC-MS/MS analysis. (**A**) Peptide hit count and (**B**) intensity sum per peptide residue. Peptides with five and more residues were analyzed through PEAKS studio through the UniProt database. The dotted line indicates the difference between the two analyses. (**C**) Hit count and (**D**) intensity sum of peptides with potential antioxidant activity based on structural requirements. Unique and common peptides ident through (**E**) Hydrophilic Interaction Liquid Chromatography (HILIC) and (**F**) Reverse Phase Chromatography (RPC).

The sample EWPN had a higher number of peptides in the range of 5–8 amino acids than EWPA (Figure 4A). The intensity distribution for these samples follows a similar pattern in which the intensity sum for sample EWPN is two-fold higher than its EWPA counterpart for 5 and 9 amino acid residue peptides. EWPA had a higher intensity for longer peptides with 33 amino acid residues (Figure 4B). The peptide hits and intensity sum obtained from the peptides that comply with constraints of the antioxidant properties were higher on EWPN samples compared to EWPA (Figure 4C,D). This could also be influenced by the higher number of peptides and intensity present in the EWPN sample. Therefore, to consider this influence, a percentage of the peptides with the antioxidant properties were calculated by the number of hits from the total amount of peptides detected through the HILIC and RPC, respectively (data not shown).

In HILIC analysis, the sample EWPN had 20% percent of peptides complying with this characteristic, while EWPA had 17%. Nonetheless, in RPC analysis, EWPA had a higher percentage of peptides complying with the antioxidant properties with 16% compared to 13% in EWPN. These results possibly explain the results obtained earlier in the present study. The observed differences in gastric and intestinal digestibility (Figure 2) of the heat-treated (60 °C) EWPA and EWPN samples could result in different populations of peptides. With higher gastric digestibility, GI digestion of EWPA resulted in larger peptides with antioxidant activity, while digestion of EWPN, with higher intestinal digestibility, resulted in smaller peptides with antioxidant activity.

The Venn diagram in Figure 4E,F showed a higher number of unique peptides for EWPN through both HILIC and RPC. Additionally, 117 and 278 common peptides were identified between EWPA and EWPN by HILIC and RPC. The average intensity among the common peptides was 2 × 10^7^ for EWPA and EWPN in HILIC analysis and 5 × 10^7^ for EWPA and 7 × 10^7^ for EWPN in RPC analysis. The intensity score of each peptide was normalized by the respective samples’ average intensity score to calculate the fold change. A total of 14 peptides from the pool of 117 common peptides after HILIC analysis (Figure S1) and 32 peptides from the pool of 278 common peptides after RPC analysis (Figure S2) were identified with the fold change >2 in EWPA and EWPN samples. The principal component analysis (PCA) showed all the variation between peptide intensity comes from the type of egg white with 100% of the variation explained by principal component 1 (data not shown). Therefore, the intensity of small molecular weight peptides between both samples differs. The unique peptides and the peptides with higher intensity identified in these two samples could be responsible for observed antioxidant activities. However, further detailed studies are required to identify the most potent sequence or synergistic relationship among several potent peptides that may be responsible for the observed antioxidant effect.

Additionally, the results suggest that the egg white processing affects the peptide intensity obtained after heat treatment at 60 °C and gastrointestinal digestion. The influence of ovalbumin aggregated morphology on in vitro simulated gastric and intestinal digestion has been studied through the PCA and cluster analysis [41]. It was suggested that peptic cleavages were favored by ovalbumin aggregation. It has also been reported that EWPA form water-soluble aggregates, suggesting a possible relationship of ovalbumin aggregation in EWPA samples and increased gastric degree of hydrolysis observed in the present study in Figure 2A at 60 °C.

These results provide the foundation for further experiments, which should consider a more profound understanding of pH and heat treatment’s influence on the peptide profile after gastrointestinal digestion and their respective biological activities.

## 4. Conclusions

In the present study, the digestibility of egg white powders treated at different heating temperatures was studied by in vitro digestion followed by the digests’ analysis. The pH of EWP had a direct effect on the denaturation and solubility of proteins. Heating of EWP might have resulted in aggregation of proteins, which may cause loosening of intermolecular bonds and enhance the digestive enzymes’ accessibility. However, further analysis is required to delineate the effect of heat treatment on the egg white proteins’ structure. EWPA (60 °C) showed better gastric digestibility and antioxidant activity than the control sample (20 °C). The GI digest of EWP (60 °C) subjected to cellular antioxidant activity analysis exhibited better antioxidant activity at neutral pH than alkaline pH only at low dosage, which might be due to the higher presence of the smaller peptides in EWPN with potential antioxidant activity. Future studies should consider the effect of EWP hydrolysate on cell proliferation to determine the effect of long-term exposure. The peptides’ identification and characterization through LC-MS/MS confirmed the antioxidant activity mechanism was based on the presence and intensity of low molecular weight peptides in the Caco-2 cell model modulated by the EWP processing. Therefore, the study concludes that heat treatment at 60 °C can improve the digestibility of egg white proteins and could potentially enhance the release of peptides with potential antioxidant activity.

## Supplementary Materials

The following are available online at https://www.mdpi.com/2076-3921/9/11/1114/s1, Figure S1. EWPA and EWPN common peptide with intensity fold change > 2, obtained through de novo sequencing of Hydrophilic Interaction Liquid Chromatography (HILIC). Figure S2. EWPA and EWPN common peptide with intensity fold change > 2, obtained through Reverse Phase Chromatography (RPC). Table S1. Composition of simulated digestion fluids for a 1.25× concentration (400 mL).
