# Supplementary material for: Effect of pH and Heat Treatment on the Antioxidant Activity of Egg White Protein-Derived Peptides after Simulated In-Vitro Gastrointestinal Digestion
Source: Antioxidants (Basel). 2020 Nov 11;9(11):1114. doi: 10.3390/antiox9111114 (PMC7697783; doi:10.3390/antiox9111114)

Supplementary Material

Effect of pH and Heat Treatment on the Antioxidant Activity of Egg white Protein-derived Peptides After Simulated In-vitro Gastrointestinal Digestion

Priyanka Singh Rao[a], Emerson Nolasco[b], Akihiro Handa[c], Michael J. Naldrett[d], Sophie Alvarez[d] and Kaustav Majumder[b*]

[a] Dairy Chemistry Division, National Dairy Research Institute (ICAR-NDRI), Karnal, Haryana 132001, India
[b] Department of Food Science and Technology, University of Nebraska-Lincoln, Lincoln, NE, 68588-6205, United States
[c] Institute of Technology Solution, R&D Division, Kewpie Corporation, 2-5-7 Sengawa, Chofu, Tokyo, 1820002 Japan,
[d] Proteomics and Metabolomics Facility, Nebraska Center for Biotechnology, University of Nebraska-Lincoln, Lincoln, NE, 68588-6205, United States

* **Correspondence:** kaustav.majumder@unl.edu; Tel: (402)-472-3510; Fax: (402) 472-4474;

Table S1. Composition of simulated digestion fluids for a 1.25X concentration (400 mL).

Salt solution added	SSF (pH 7)		SGF (pH 3)		SIF (pH 7)	
	Vol. of stock added to make 400mL of 1.25X	Final salt conc. in SSF	Vol. of stock added to make 400mL of 1.25X	Final salt conc. in SGF	Vol. of stock added to make 400mL of 1.25X	Final salt conc. in SIF
	(mL)	(mM)	(mL)	(mM)	(mL)	(mM)
KCl	15.1	15.1	6.9	6.9	6.8	6.8
KH_2PO_4	3.7	3.7	0.9	0.9	0.8	0.8
NaCl	2.3	13.6	12	72.2	20.6	123.4
$MgCl_2$	0.2	0.15	0.1	0.1	0.3	0.33
$(NH_4)_2CO_3$	0.1	0.06	0.5	0.5	-	-
HCl (1M)	-	-	-	-	-	-
$CaCl_2(H_2O)_2$[b]	-	-	-	-	-	-
Water	378.6	-	379.6	-	371.5	-

Note: The volume of 1M NaOH or HCl used to adjust the pH of the simulated digestion fluid was subtracted from the water volume to make up the volume to 400 mL. SSF: Simulated salivary fluid, SGF: Simulated gastric fluid, SIF: Simulated intestinal fluid, conc.: concentration (Mat, Cattenoz, Souchon, Michon, & Le Feunteun, 2018; Minekus et al., 2014).

References

Mat, D. J. L., Cattenoz, T., Souchon, I., Michon, C., & Le Feunteun, S. (2018). Monitoring protein hydrolysis by pepsin using pH-stat: In vitro gastric digestions in static and dynamic pH conditions. *Food Chemistry*, *239*, 268–275. https://doi.org/10.1016/j.foodchem.2017.06.115

Minekus, M., Alminger, M., Alvito, P., Ballance, S., Bohn, T., Bourlieu, C., … Brodkorb, A. (2014). A standardised static in vitro digestion method suitable for food-an international consensus. *Food and Function*, *5*(6), 1113–1124. https://doi.org/10.1039/c3fo60702j

Figure S1. EWPA and EWPN common peptide with intensity fold change > 2, obtained through de novo sequencing of Hydrophilic Interaction Liquid Chromatography (HILIC).

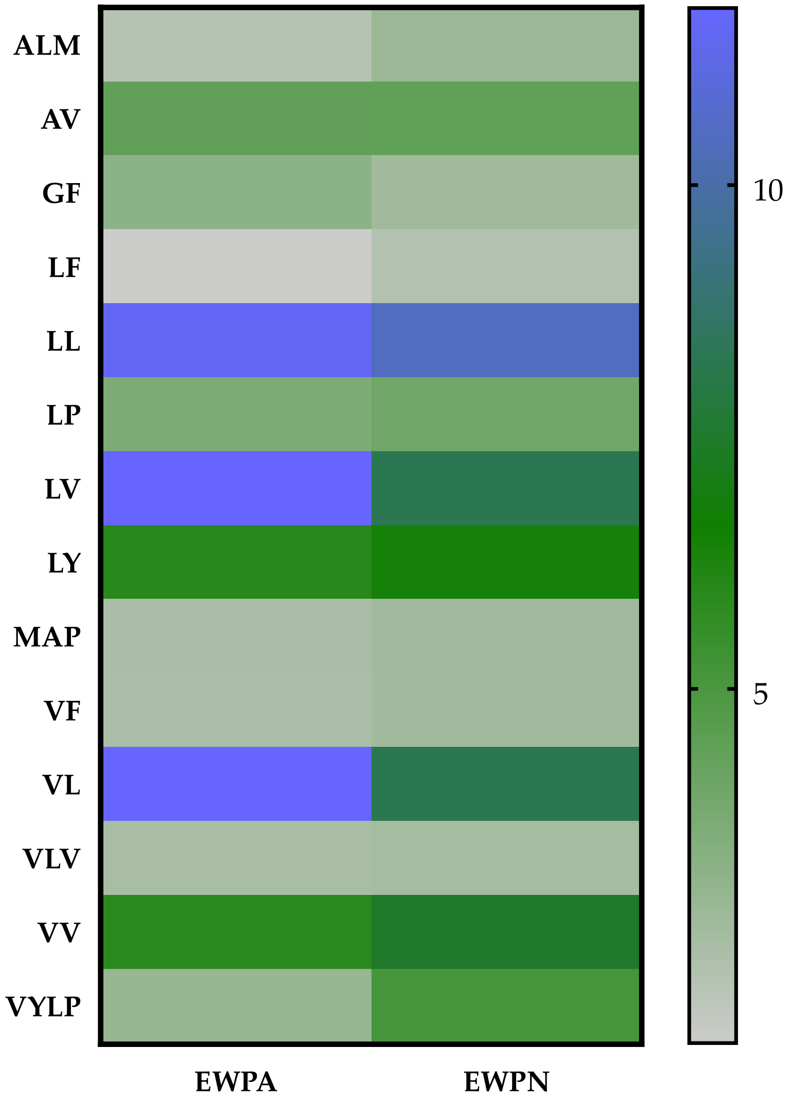

Figure S2. EWPA and EWPN common peptide with intensity fold change > 2, obtained through Reverse Phase Chromatography (RPC).

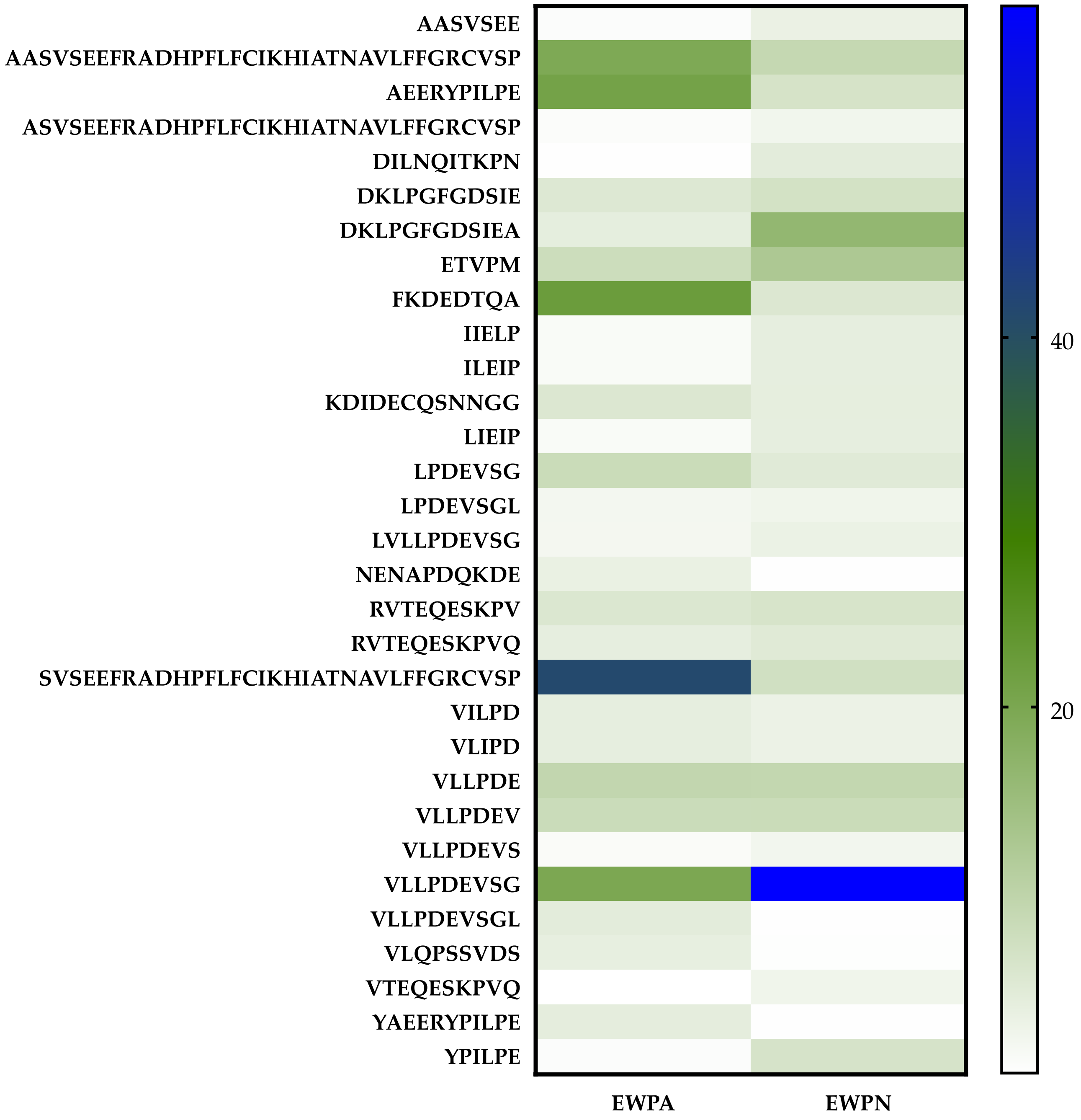

Supplement: Supplementary file 1 [file antioxidants-09-01114-s001.pdf]
